# Direct Ethanol Production from Lignocellulosic Sugars and Sugarcane Bagasse by a Recombinant *Trichoderma reesei* Strain HJ48

**DOI:** 10.1155/2014/798683

**Published:** 2014-06-02

**Authors:** Jun Huang, Dong Chen, Yutuo Wei, Qingyan Wang, Zhenchong Li, Ying Chen, Ribo Huang

**Affiliations:** ^1^State Key Laboratory for Conservation and Utilization of Subtropical Agro-Bioresources, Guangxi University, 100 Daxue Road, Nanning, Guangxi 530004, China; ^2^College of Life Science and Technology, Guangxi University, Nanning 530004, China; ^3^National Engineering Research Center for Non-Food Biorefinery, Guangxi Academy of Sciences, 98 Daling Road, Nanning, Guangxi 530007, China

## Abstract

*Trichoderma reesei* can be considered as a candidate for consolidated bioprocessing (CBP) microorganism. However, its ethanol yield needs to be improved significantly. Here the ethanol production of *T. reesei* CICC 40360 was improved by genome shuffling while simultaneously enhancing the ethanol resistance. The initial mutant population was generated by nitrosoguanidine treatment of the spores, and an improved population producing more than fivefold ethanol than wild type was obtained by genome shuffling. The results show that the shuffled strain HJ48 can efficiently convert lignocellulosic sugars to ethanol under aerobic conditions. Furthermore, it was able to produce ethanol directly from sugarcane bagasse, demonstrating that the shuffled strain HJ48 is a suitable microorganism for consolidated bioprocessing.

## 1. Introduction

Lignocellulosic biomass is an abundant renewable resource and sustainable resource of biofuels, biochemicals, and biomaterials [1]. However, Lignocellulosic biomass is designed by nature to resist hydrolysis. Currently, dilute-acid and enzyme have been used initially for lignocellulosic materials degradation for biofuel. But both processes are expensive, slow, and inefficient. Therefore, consolidated bioprocessing (CBP), where the conversion of lignocellulose into desired products is carried out by one microorganism in one step, has been a subject of increased research effort in recent years [2, 3]. CBP offers the potential for lower biofuel production costs due to simpler feedstock processing, lower energy inputs, and higher conversion efficiencies than the processes based on simultaneous saccharification and fermentation (SSF). A particular challenge in the development of CBP is to pursue a perfect microorganism, which possesses all properties of lignocellulose utilization and ethanol production. Recent researches demonstrated that* T. reesei* can be considered as a potential candidate for CBP [4].

The enzyme producer* T. reesei *stands out among industrially applied microorganism, because it can degrade cellulose at the rates sufficient for industrial use. And a wide range of mutants have been developed for* T. reesei* [5]. Furthermore,* T. reesei* has the ability to utilize all the lignocellulose sugars for producing ethanol [4]. Therefore,* T. reesei* is one of the primary candidates for CBP research [6].

Genome shuffling is a useful method for rapid breeding of improved microorganisms and involves the generation of mutant strains that have an improved phenotype, followed by multiple rounds of protoplast fusion to allow recombination between genomes [7, 8]. Genome shuffling accelerates genetic changes at different positions throughout the whole genome without understanding the knowledge of detailed genetic information. However, this approach depends on the efficiency of the classic CaCl_2_/PEG-based scheme, which has the disadvantages of fusant instability and low fusion efficiency [9]. Recently, a recombinant yeast strain with enhanced xylose fermentation has been developed through genome shuffling by electroporation [10]. Nevertheless there are no further reports and applications applied to* T. reesei* so far despite the fact that this method is more convenient than the conventional protoplast fusion technique. Thus, the present study was aimed at isolating protoplasts from* T. reesei *mutant strain and carried out genome shuffling with the genome of* S. cerevisiae* by electroporation to achieve rapid improvement of ethanol production. In addition, the experiments of this study were investigated in shake-flasks.

## 2. Materials and Methods 

### 2.1. Strains

*T. reesei* CCIC 40360 was obtained from China Center of Industrial Culture Collection (CCIC), and it was maintained aerobically on Potato Dextrose Agar (PDA) agar slants.* Saccharomyces cerevisiae *CICC31279 was also obtained from CICC and was cultured on Yeast Extract Peptone Dextrose agar (YPD) slants.

### 2.2. Media and Culture Conditions

The composition of* Trichoderma* minimal medium (MM) and regeneration of protoplasts medium (RM) were prepared as described previously [11]. The growth medium (GM) contained (per liter) 4 g glucose, 5 g yeast extract, and 3 g potato extract. The fermentation medium (FM) contained 50 g glucose, 10 g yeast extract, 10 g KH_2_PO_4_, 2 g (NH_4_)_2_SO_4_, and 0.5 g MgSO_4_·7H_2_O per liter.

### 2.3. Preparation of Starting Strains for Genome Shuffling

*T. reesei* CCIC 40360 was mutagenized with nitrosoguanidine (NTG) to obtain the initial mutant library as described [12]. 1 mL spores of* T. reesei *CCIC 40360 (1 × 10^8^–5 × 10^8^) were treated with 5 mL of 0.01% NTG in 10 mM Tris-HCl (pH 9.0) buffer for 15 min. The spores were subsequently washed three times with 2 mL of 10 mM Tris-HCl (pH 9.0). After appropriate dilution, the suspension of spores was spread on the MM agar plates containing 3% (v/v) ethanol, on which the wild-type CCIC 40360 could not exist. The fast grown colonies were picked up for shake-flask analysis to determine their ethanol production individually.

### 2.4. Genome Shuffling

The genomic DNA of* S. cerevisiae* 31279 was extracted using a modification of the cetyltrimethylammonium bromide method [13]. Protoplast preparation of* T. reesei *was essentially done as described [11]. Genome shuffling was similar to the procedure described [10]. The electroporation was conducted by ECM630 (BTX, USA). After genome shuffling, the transformed cells were transferred to liquid regeneration medium containing 3% (v/v) ethanol and incubated at 30°C overnight on a rotary shaker at 130 rpm. Then, the cultures of regenerated protoplasts were diluted and then spread on MM plates containing 3% (v/v) ethanol and incubated at 30°C for 5-6 days. The colonies appearing under these conditions were selected to carry out shake-flask analysis and the strains with higher ethanol productivity were selected and named S1. Three rounds of genome shuffling were carried out, and after each round potential recombinant strains were used as the sources of protoplasts for the subsequent rounds of genome shuffling, which were carried out using the same methods.

### 2.5. Shake-Flask Fermentation

Fungal mycelia were grown on PDA agar plate for 7 days and then three loops of the mycelium mat were inoculated into 500 mL of GM medium in a 1 L Erlenmeyer flask. After incubation for 4 days at 180 rpm at 30°C, the mycelium was harvested (20 ± 2 g, wet weight) and transferred aseptically to a 100 mL Erlenmeyer flask containing 20 mL FM medium. FM medium without sugar was used as the control. In aerobic conditions, the Erlenmeyer flasks were covered with cotton and shaken at 130 rpm. In anaerobic conditions, the flasks were performed using controlled atmosphere chamber (plas.labs, MI, USA) at 30°C.

### 2.6. Random Amplified Polymorphic DNA (RAPD)

DNA was isolated from parent strain and its mutants by a procedure described in [13]. RAPD amplification was performed in a buffer (50 *μ*L) which contained 5 *μ*L of 10x PCR buffer, 3 *μ*L of MgCl_2_ (25 mM), 1 *μ*L of dNTP mixture (2.5 mM), 5 U of Taq DNA polymerase, 25 ng of template DNA, 2 *μ*L of primer (Table 1), and 36 *μ*L of ddH_2_O. All these reagents were purchased from Takara (Japan). Amplification was run in a Biometra thermocycler (Biometra, Germany) set at the following program: 95°C for 5 min, followed by 45 cycles of 95°C for 30 s, 36°C for 1 min, and 72°C for 2 min. After that, a 10 min final extension at 72°C was conducted to stabilize the amplified DNA products. Such amplified products were separated by electrophoresis in 1.0% agarose gel and visualization in a UV transilluminator.

### 2.7. Mill Treatment of Sugarcane Bagasse

Sugarcane bagasse was dried in an oven at 60°C until the weight was constant. The dry sugarcane bagasse was milled in a rotary mill (Thomas Wiley model 4, USA) and passed through a 1.0 mm screen. The milled sugarcane bagasse was used for subsequent experiments as a substrate for fermentation.

### 2.8. Enzyme Assays

Endoglucanase activity (carboxymethyl cellulase activity) in the culture supernatant was determined as described by Mandels et al. [16]. FPase activity (filter paper activity) was determined by the method of Ghose [17]. Units (IU) of FPase, and endoglucanase were defined as the 1 *μ*mol of glucose equivalent liberated per minute under assay conditions. The released sugar was measured by the dinitrosalicylic acid method [18]. The released sugar was measured by the dinitrosalicylic acid method.

### 2.9. Analytical Methods

The ethanol concentration was analyzed by GC (model N5690; Agilent Technologies Inc.). The concentrations of residual sugar in fermentation broths were analyzed by HPLC with an Aminex-87H column (Bio-Rad, Hercules, CA) maintained at 60°C.

## 3. Results 

### 3.1. Selection of Starting Strains for Genome Shuffling

Genome shuffling requires a diverse population of mutants that already show some improvement in the trait of interest compared with that in the initial strain. In this work, NTG mutation was used to generate populations of mutants of CCIC 40360. After the spores were treated with NTG, they were 20 NTG mutant strains selected from MM plates containing 3% (v/v) ethanol, on which the wild-type CCIC 40360 could not exist. During the subsequent screening in shake-flask evaluations, NTG 1 was shown better than other mutants (data not shown). Although glucose was consumed completely within 96 h, NTG 1 exhibited further improved ethanol production (3 ± 0.1 g/L) than CCIC 40360 (2 ± 0.1 g/L) after 120 h cultivation. Consequently, strain NTG 1 was used as the starting population for genome shuffling.

### 3.2. Genome Shuffling of Improved Mutant Population

**Figure 1:**
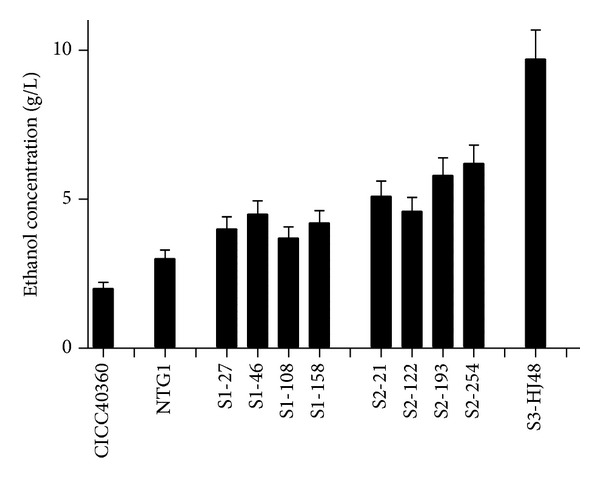
Improvement of ethanol yield by genome shuffling. One to three rounds of genome shuffling were used to improve ethanol yield of* Trichoderma reesei* CICC 40360. S1: the first round of genome shuffling; S2: the second round of genome shuffling; S3: the third round of genome shuffling. The bars represent mean ethanol yield with less than 10% standard deviation.

In this study, unlike the case in unicellular yeasts, the freshly prepared protoplasts were used for electroporation. The protoplast of NTG1 was used as the starting population for genome shuffling. Three successive rounds of genome shuffling were carried out. After the first electroporation, 200 colonies were randomly selected on plates containing 3% ethanol and were further assayed for ethanol production in FM-liquid culture. We found four isolates (S1-27, S1-46, S1-108, and S1-158) that exhibited further improved productivity of ethanol among 200 colonies (4 ± 0.2, 4.5 ± 0.2, 3.7 ± 0.2, and 4.2 ± 0.2 g/L, resp., Figure 1). These isolates were used as the population for the second round of genome shuffling.

After the second electroporation, 15 colonies were obtained on plates containing 3.5% ethanol and were screened for their ethanol productivity. Another four colonies were selected from this round and used for the next round of genome shuffling. These four isolates (S2-21, S2-122, S2-193, and S2-254) could produce more ethanol than other S2 strains did in shake flasks (5.1 ± 0.2, 4.6 ± 0.2, 5.8 ± 0.2, and 6.2 ± 0.2 g/L, resp., Figure 1). After the third electroporation, 10 colonies were obtained on the plates containing 4% (v/v) ethanol. The best performing shuffled strain from the third round, HJ48, which had higher ethanol production capacity (9.7 ± 0.2 g/L) after 96 h cultivation, was selected for further studies (Figure 1). As a control, the protoplast of mutant NTG1 was treated by five successive rounds of NTG mutagenesis. We found that no colony appeared on the agar plates containing 4% ethanol and obviously improved ethanol production. This result indicated that the shuffled strain HJ48 can be achieved only by successive rounds of genome shuffling.

As a control, the protoplast of mutant NTG1 was treated by five successive rounds of NTG mutagenesis. We found that no colony appeared on the agar plates containing 4% ethanol and obviously improved ethanol production. This result indicated that the shuffled strain HJ48 can be achieved only by successive rounds of genome shuffling. Thus, compared to the traditional protoplast fusion techniques, our genome shuffling method has the advantages of high efficiency and easy operation. We first successfully applied this genome shuffling method to construct a recombinant filamentous fungi strain with enhanced ethanol production.

### 3.3. RAPD Analysis to Identify Genomic Variation in the Course of Genome Shuffling

**Figure 2:**
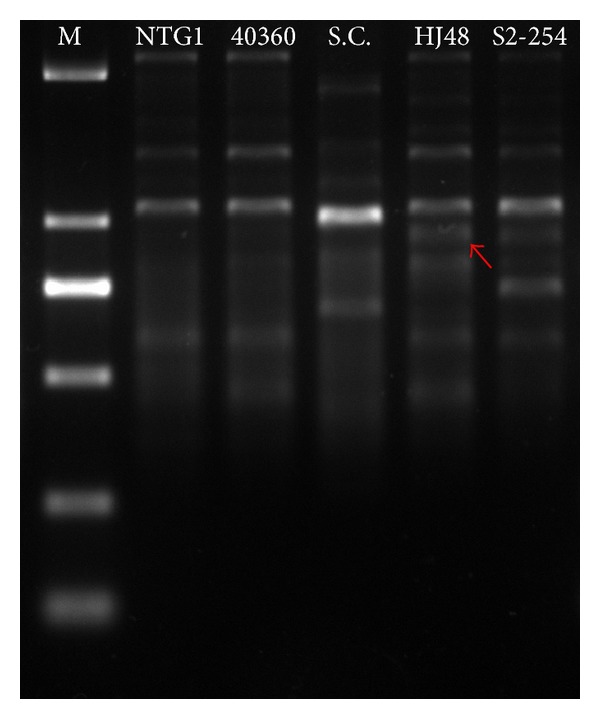
Genetic variation of fungus by Random Amplified Polymorphic DNA (RAPD) analysis. (Lane 1: marker; Lane 2; mutant NTG1; Lane 3:* T. reesei* CICC 40360S2-254; Lane 4:* S. cerevisiae* 31279; Lane 5; HJ48; Lane 6: S2-254.).

To confirm genome shuffling, an RAPD polymorphism analysis was carried out using the wild-type* T. reesei *CICC40360,* S. cerevisiae* 31279, and the shuffled strains. Using P13 (sequence GTCCACTGTG) as primer, a large number of DNA bands were obtained from the templates of the recombinant fungi strain genomes. Differences were clearly observed between the RAPD profiles of the parents and shuffled strains (Figure 2).

### 3.4. Ethanol Production in Glucose-Based FM Medium

**Figure 3:**
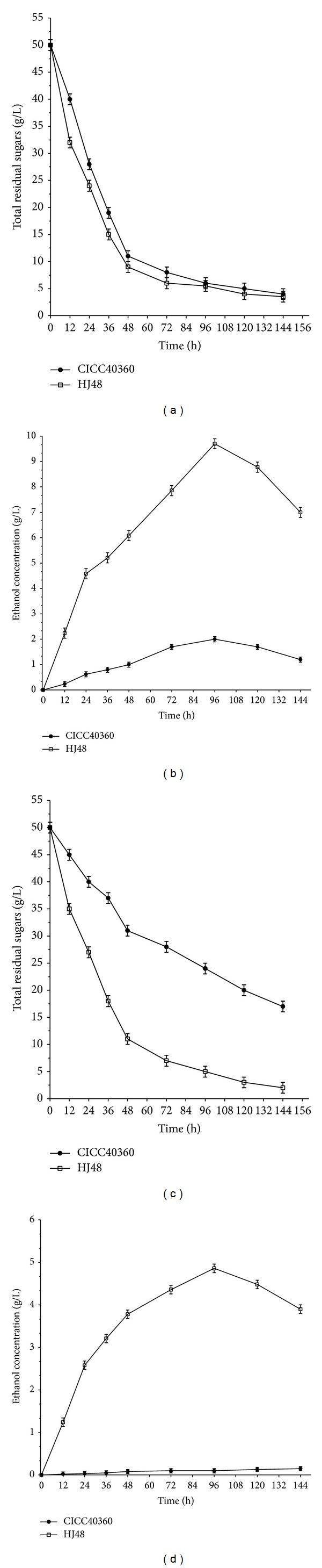
Time courses of glucose consumption and ethanol production by HJ48 and CICC40360 under aerobic conditions (a) and (b) and anaerobic conditions (c) and (d).

In general, anaerobic conditions are better than aerobic conditions for the production of ethanol by alcohol-fermenting microorganisms. Nevertheless, under anaerobic condition,* T. reesei* produces ethanol at a cost inefficient rate [4]. In this study, the ethanol-producing performances of HJ48 and CICC40360 were examined under both aerobic and anaerobic conditions (Figure 3). HJ48 produced ethanol under anaerobic conditions, but the yield was lower than under aerobic conditions. The maximum ethanol yield was 9.7 ± 0.2 g/L after 96 h cultivation under aerobic conditions, whereas the maximum yield under aerobic conditions after 96 h cultivation was 4.8 ± 0.2 g/L. This result indicated that HJ48 can effectively convert glucose to ethanol under aerobic conditions and produce ethanol at comparable levels to other fungal species [14, 19] (Table 2).

In contrast to HJ48, glucose was negligibly converted to ethanol by CICC40360 under both aerobic and anaerobic conditions. CICC40360 was cultured under aerobic conditions, showing maximum ethanol concentrations of 2.1 ± 0.1 g/L after 96 h cultivation. In contrast, under anaerobic conditions, the ethanol produced by CICC40360 was negligible.

### 3.5. Ethanol Production Using Various Carbon Sources

In order to examine the fermentation ability of CCIC 40360 and HJ48 to consume others hexoses and pentoses under aerobic conditions, glucose was replaced by galactose, fructose, mannose, cellobiose, xylose, and arabinose in FM media. The results of these experiments are presented in Table 3. In contrast to HJ48, the ethanol production of CCIC 40360 was significantly lower under the same conditions. In the present study, the maximum ethanol concentration by HJ48 using these sugars was reached after 6 ± 2 days. The ethanol yields using these different hexoses were lower than the yield produced with glucose. The conversion of the pentoses to ethanol, xylose, and arabinose was less efficient. The ethanol titer obtained by HJ48 was higher than those obtained by other fungus (Tables 3 and 4). The result demonstrated that HJ48 can convert all biomass sugars to ethanol under aerobic conditions; however, the ethanol yields and production rates are low. Further studies are required to improve the bioethanol yield and productivity of this microorganism.

### 3.6. Direct Ethanol Production from Sugarcane Bagasse

**Figure 4:**
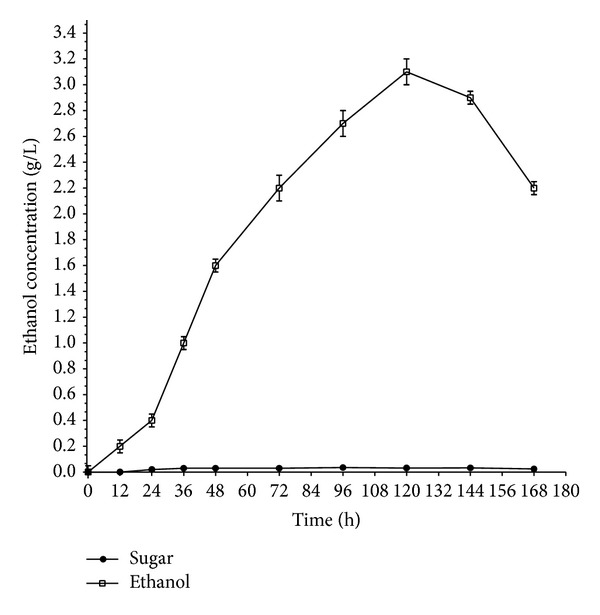
Time course of ethanol production by HJ48 and CICC40360 using 50 g/L sugarcane bagasse as the sole carbon source.

HJ48 was cultured in FM medium containing sugarcane bagasse as a typical lignocellulosic material to further characterize the fermentation properties of this shuffled strain. The sugarcane bagasse used in this study contains 35.63% cellulose, 26.88% hemicellulose, 24.31% lignin, 5.29% ash, and 7.89% of other components. HJ48 cultured in 50 g/L sugarcane bagasse yielded a maximum ethanol concentration of 3.1 ± 0.2 g/L after 120 h cultivation (Figure 4). Considering the cellulose and hemicellulose fraction of the sugarcane bagasse, the ethanol yields per gram of biomass were 0.10 g/g. In this case, the liberated glucose was detected in the culture during fermentation, indicating that lignocellulosic had decomposed gradually. As expected, CICC40360 could not convert sugarcane bagasse to ethanol. There is no report about direct ethanol production from sugarcane bagasse without pretreatment under aerobic condition until now (Table 5).

At the same time, the hydrolysis efficiency of the HJ48 using sugarcane bagasse was also investigated (data not shown). After 120 h of the incubation time, the maximal FPase and endoglucanase activity were obtained for HJ48 (0.34 and 3.25 IU/mL, resp.), which were 1.8 and 2.1-fold higher than the parent strain (0.19 and 1.55 IU/mL, resp.).

In the present study, our data clearly demonstrated that HJ48 had the saccharification and fermentation ability towards cellulosic materials under aerobic condition. The production of ethanol from milled sugarcane bagasse by HJ48 without pretreatment has the potential to be developed into a cost effective process for producing bioethanol from lignocellulosic materials such as rice, straw, and wheat bran. Further study into the fermentation ability of HJ48 is needed by examining the suitability of various cellulosic materials and conditions.

### 3.7. The Genetic Stability of the HJ48

To check the genetic stability of HJ48, the shuffled mutants from three successive rounds of genome shuffling were cultured for 20 generations and the ethanol tolerance and ethanol production of every other generation were measured. All the generations showed similar tolerance and production as the initial strain suggesting that HJ48 was genetically stable and suitable for the next investigation.

## 4. Discussion

Improvement of industrial strains for overproduction of the target bioproducts plays an important role in industrial applications. Traditional metabolic engineering was effective in improving phenotypes of* T. reesei* strains for cellulase production; it normally involves the constitutive expression of multiple genes followed by necessary mutagenesis and postevolutionary engineering [5]. With the broad application of recombinant DNA technology, novel methods and strategies are exploited for engineering single gene, pathways, and even whole genomes of the industrial* T. reesei* strains, such as genome shuffling [4, 7]. In the present study, we successfully combined genome shuffling and mutagenesis to significantly improve production of ethanol in* T. reesei*.

As a single* T. reesei* strain, CICC40360 was the starting point of the evolution program, an improved population was required for genome shuffling. Classical method such as NTG mutagenesis was sufficient to generate improved populations of genetically diverse strains, with slight improvements in ethanol production (Figure 1). Genome shuffling of these populations by three rounds of genome shuffling generated a new population of strains with further improvements in ethanol tolerance and ethanol production (Figure 1); the third shuffled population (F3) contained members that could grow on RM plates containing 4% ethanol. The successive improvement of populations that had undergone successively more recombination (F3 > F2 > F1; Figure 1) illuminates the importance of recombination in the improvement process.

Genome shuffling uses recursive genetic recombination through protoplast fusion. This strategy was successfully applied in rapid strain improvement of both prokaryotic and eukaryotic cells [7, 8]. However, this method largely depends on the efficiency of the traditional protoplast fusion techniques, which have the disadvantages of fusant instability and low fusion efficiency [9]. In this study, we attempted to construct a recombinant fungi strain using a modified genome shuffling method. Instead of using recursive protoplast fusion, recursive direct genome isolation and transformation were used for gene recombination. The improved method shares the same advantages with the protoplast fusion-based genome shuffling method for rapid complex phenotype improvement. In addition, it is time-saving, easier to operate, and has higher gene recombination efficiency.

Successful works on selection of fungi for their ability to produce ethanol have been reported by several investigators. For example, Xu et al. analyzed the potential of* T. reesei* as CBP organism [4]. They selected three strains of* T. reesei* capable of producing ethanol from lignocellulosic sugars and cellulose under anaerobic conditions. However, they did not report any details of fermentation. Okamoto et al. found that* P. cinerea* and* T. suaveolens *efficiently produced ethanol [14]. The maximum ethanol yields obtained from 20 g/L glucose were 6.7 and 1.3 g/L using* P. cinerea* and* T. suaveolens* under aerobic conditions, respectively. However, the ethanol produced from cellulose by* P. cinerea* was 3 g/L after 18 days of being cultured. de Almeida et al. isolated* F. verticillioides* and* A. zeae* that could produce ethanol directly from sugars and pretreatment sugarcane bagasse [15]. Unfortunately, the process of pretreatment sugarcane bagasse requires significant amounts of energy. In our study, we pretreated sugarcane bagasse by using a rotary mill, which is an environment-friendly treatment compared with sodium hypochlorite treatment. Considering energy consumption costs, direct ethanol production from lignocellulosic biomass using* T. reesei* as a biocatalyst is an efficient and economical process, as it requires no pretreatment such as sodium hypochlorite treatment or acid hydrolysis.

To minimize the number of screens required for selecting improved strains, we incorporated an ethanol resistant mutant isolation step into the method. In the traditional study, the protoplast regeneration applied in genome shuffling was carried out on agar plate. But, as is already known, ethanol is easy to evaporate. According to the literature, the liquid method of protoplast regeneration is an attractive process to greatly increase the frequency of protoplast regeneration compared with the agar method [20]. To facilitate the precision of genome shuffling procedures, in our experiment, we used liquid RM to regenerate protoplast to prevent ethanol volatilization. The result demonstrates that this method could reduce time for screening fusants and improve work efficiency.

This is the first report on the fermentation performance of the fungi* T. reesei *under aerobic conditions, where genome shuffling method was used to improve ethanol production and ethanol tolerance in* T. reesei*. With the development of the tools described in this report, a shuffled strain HJ48 was obtained, which shows efficient fermentation of various carbon sources under aerobic condition. Furthermore, the shuffled strain HJ48 can tolerate 4% (v/v) ethanol stress and also produce ethanol directly from sugarcane bagasse, indicating that it is a promising microorganism for application in consolidated bioprocessing of lignocellulosic biomass.

## Figures and Tables

**Table 1:** Primers used for random amplified polymorphic DNA.

Primer	Sequence (5′ to 3′)
P1	GTTGGTGGCT
P2	ACAACGCCTC
P3	GGGGGATGAG
P4	GGCGGTTGTC
P5	GGGAACGTGT
P6	CTGGGCAACT
P7	CCGTGACTCA
P8	TCTGTTCCCC
P9	GTCTTGCGGA
P10	TCTGGCGCAC
P11	GTCCACTGTG
P12	GGGACGTTGG
P13	GGTGGTCAAG
P14	AGGGTCGTTC
P15	GACCTACCAC
P16	GTAACCAGCC
P17	TCAGTCCGGG
P18	CACCATCCGT
P19	CCTTCAGGCA
P20	AGGTCTTGGG

**Table 2:** Ethanol production by fungi grown on glucose under aerobic condition.

Fungus	*S* (g/L)	Control	*Y*_ME_ (g/g)	Reference
*T. reesei *CICC40360	50	—	0.042^a,b^	This study
*T. reesei *HJ48	50	—	0.21^a,b^	This study
*P. cinerea *	20		0.33	[14]
*T. suaveolens *	20		0.065	
*F. verticillioides *	20		0.07	[15]
*A. zeae *	20		0.05	

*S*: substrate concentration; *Y*_ME_: yield of metabolized ethanol (consumed sugar); —: no production.

^a^The displayed values are the average of three independent experiments.

^b^The value was determined by growing through the two-stage culture process using preculture.

**Table 3:** Fungus producing ethanol from biomass directly.

Organism	Number of tested strains	FC	Glucose (g/L)	Mannose (g/L)	Galactose (g/L)	Fructose (g/L)	Cellobiose (g/L)	Xylose (g/L)	Arabinose (g/L)	Reference
CICC 40360	1	A	2.0 ± 0.01	1 ± 0.01	0.4 ± 0.01	0.3 ± 0.01	0.1 ± 0.01	0.1 ± 0.01	—	This study
HJ48	1	A	9.7 ± 0.2	8.0 ± 0.2	2.8 ± 0.1	2.3 ± 0.1	2.5 ± 0.1	2.1 ± 0.1	1.1 ± 0.1	This study
*T. reesei *	3	AN	4.0–4.8	4.2–4.5	3–3.5			0.4-0.5	0.2	[4]
*F. verticillioides *	1	A	1.4					0.88		[15]
*A. zeae *	1	A	0.9					0.48		[15]

—: no production; FC: fermentation condition; A: aerobic; AN: anaerobic.

**Table 4:** Fungus producing ethanol from sugar directly.

Organism	FC	Glucose (g/g)	Mannose (g/g)	Galactose (g/g)	Fructose (g/g)	Cellobiose (g/g)	Xylose (g/g)	Reference
HJ48	A	0.25 ± 0.1	0.20 ± 0.1	0.18 ± 0.1	0.12 ± 0.1	0.21 ± 0.1	0.15 ± 0.1	This study
*F. verticillioides *	A	0.07					0.13	[15]
*A. zeae *	A	0.05					0.08	[15]

FC: fermentation condition; A: aerobic; g/g: indicates the g of ethanol per g of consumed sugar.

**Table 5:** Fermentation performance of diverse microorganisms using sugarcane bagasse.

Microorganism	Pretreatment	*S* (g/L)	FC	*Y*_ME_ (g/g)	Reference
*T. reesei* HJ48	Mill	50	A	0.10^a,b^	This study
*F. verticillioides *	Dilute alkali	40	OL	0.15	[15]
*A. zeae *	Dilute alkali	40	OL	0.13	[15]

^a^The displayed values are the average of three independent experiments.

^b^The value was determined by growing through the two-stage culture process using pre-culture.
